# Congenital nasal pyriform aperture stenosis: diagnosis and management

**DOI:** 10.1186/1824-7288-38-28

**Published:** 2012-06-26

**Authors:** Enrico Sesenna, Massimiliano Leporati, Bruno Brevi, Gabriele Oretti, Andrea Ferri

**Affiliations:** 1 Head and Neck Department (Head : Professor Enrico Sesenna); Maxillo-Facial Surgery Division, University Hospital of Parma, Via Gramsci 14, Parma 43120, Italy; 2 Head and neck Department (Head: Professor Teore Ferri), Otolaryngology Head and Neck Surgery Division, University Hospital of Parma, Parma, Italy

**Keywords:** Congenital nasal pyriform aperture stenosis, Sublabial approach, Newborns respiratory distress

## Abstract

**Background:**

Congenital nasal pyriform aperture stenosis is a rare and potentially lethal form of airway obstruction in newborns. Immediate recognition and appropriate therapy are mandatory for this potentially life-threating condition. This anomaly may present as an isolated malformation or may be associated with other cranial-facial anomalies. Clinically, CNPS shows unspecific symptoms of nasal airway obstruction such as apnoic crisis, episodic cyanosis and inability to nurse. The purpose of this report is to present author's experience in the surgical management of this rare pathology in 3 patients.

**Patients and Methods:**

Three cases of congenital nasal pyriform aperture stenosis were reviewed for presentation of the disorder, management and effectiveness of the surgical treatment.

**Results:**

All the patients underwent a surgical correction of the pyriform aperture stenosis using a sublabial approach followed by nasal stenting. During the follow-up no cases of restenosis, respiratory failure or cyanosis were detected.

**Conclusions:**

Congenital pyriform aperture stenosis should be suspected in newborns with clinical signs of severe nasal obstruction associated with a difficulty to pass a small catheter though the anterior nasal valve. Timely recognition is mandatory to prevent a potential deadly outcome. Surgical correction of the stenosis though a sublabial approach followed by a nasal stenting revealed to be most effective treatment for these patients.

## Introduction

Congenital nasal pyriform stenosis (CNPS) is an unusual, potentially lethal form of neonatal nasal obstruction [1]. The pyriform aperture is the most anterior and narrow opening of the bony nasal airways. It is limited laterally by the nasal process of the maxilla, inferiorly by the junction of the horizontal process of the maxilla and the anterior nasal spine, and superiorly by the nasal bones [2]. Congenital stenosis of this anatomic region is a rare condition that may present as an isolated malformation or may be associated with other craniofacial anomalies.

Clinically, CNPS manifests as nonspecific symptoms of nasal airway obstruction, such as apneic crisis, episodic cyanosis, and inability to nurse. Because infants are obligatory nasal breathers until 6–8 weeks of age, any form of nasal airway obstruction could cause severe distress symptoms; thus, early diagnosis and treatment are mandatory [3,4].

The purpose of this report is to present the author’s experience in the surgical management of this rare pathology in three patients.

## Patients and methods

### Case 1

**Figure 1:**
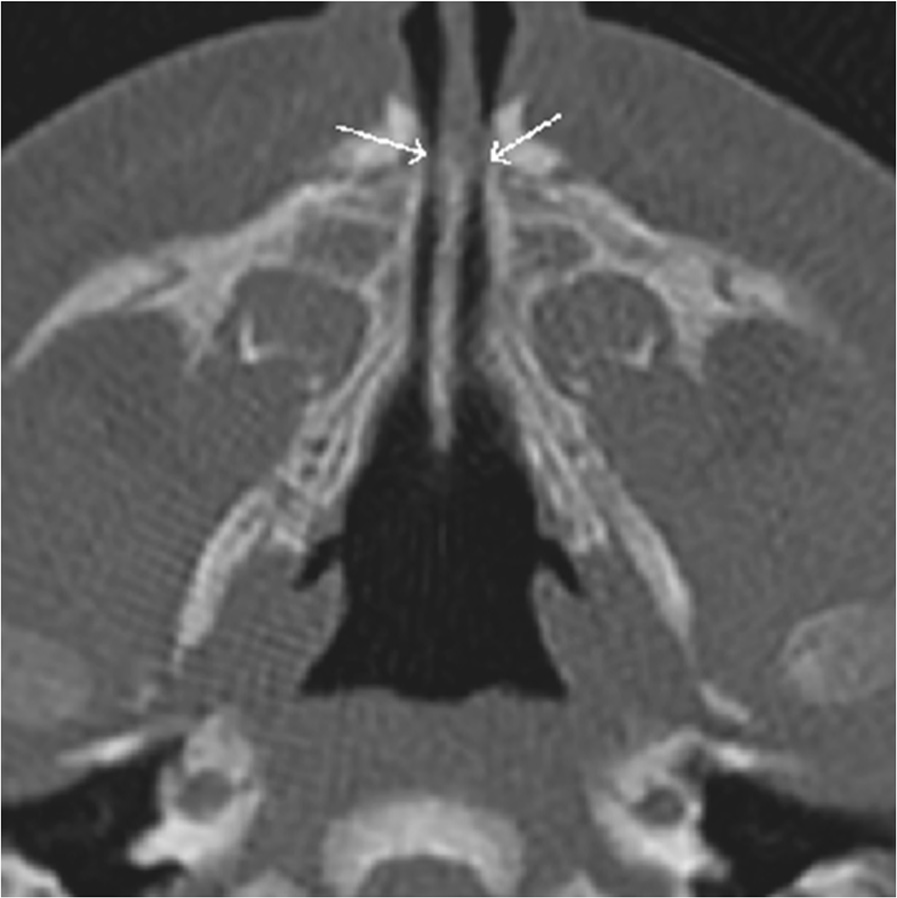
Pre-operative CT scan showing the nasal pyriform aperture stenosis.

A full-term male infant had Apgar scores of 6 at 1 min and 9 at 5 min after birth. He was 2.690 kg in weight and 51 cm in height, and had a cranial circumference of 34 cm and a thoracic circumference of 32.5 cm. The infant showed mild respiratory distress, ribcage asymmetry, a reduced vesicular murmur, stridor, and diaphragmatic retraction. Anterior rhinoscopy was not possible because of bilateral stenosis of the nasal fossae and interference of the turbinates, but transoral posterior rhinoscopy showed a normal right nasal fossa and left choanal stenosis. Computed tomography (CT) revealed stenosis of the pyriform aperture (5,3 mm diameter) and choanal atresia (Figure 1).

**Figure 2:**
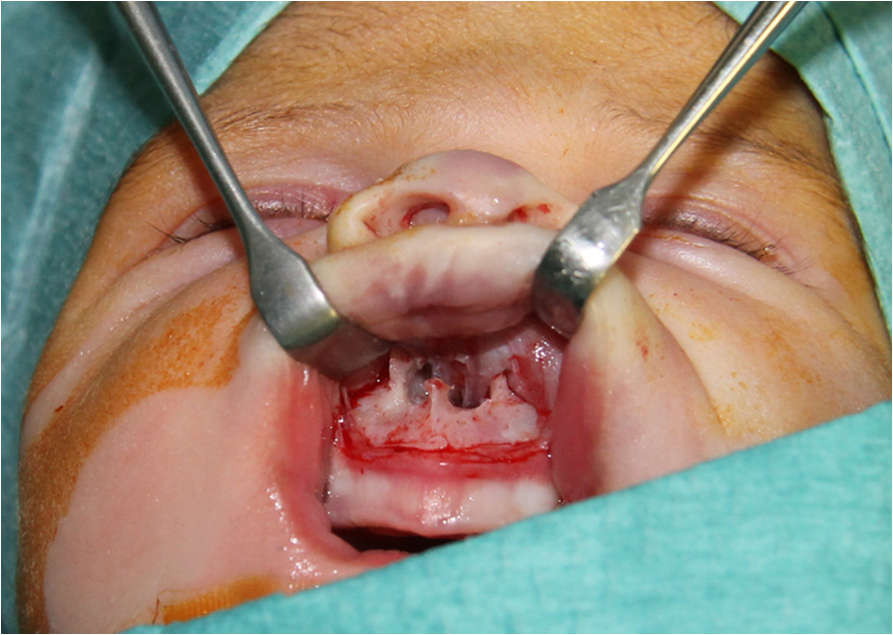
Intra-operative picture showing the enlargement of the pyriform aperture obtained with surgery.

An initial attempt of conservative treatment using nasal conformers and nasal decongestant drops failed. Given the persistence of symptoms, the infant underwent surgery at 13 days of age. Through an endo-oral sublabial approach, the stenotic pyriform area was reached with subperiosteal dissection. The aperture was widened by drilling and reshaping the bone with a 2-mm diamond bur, taking care to avoid injury to the tooth buds, contiguous soft tissues, and nasolacrimal duct. Through an endoscopic approach, the turbinates were dislocated laterally and the choanal area was enlarged by reshaping the posterior portion of the inferior turbinate and nasal mucosa. Two soft silastic nasal stents (o.d. 3.96 mm, i.d. 3.0 mm, length 29.4 mm) were sutured to the columella to maintain airway patency and stabilize the surgical results. The correct positioning of the stents was checked endoscopically (Figure 2).

**Figure 3:**
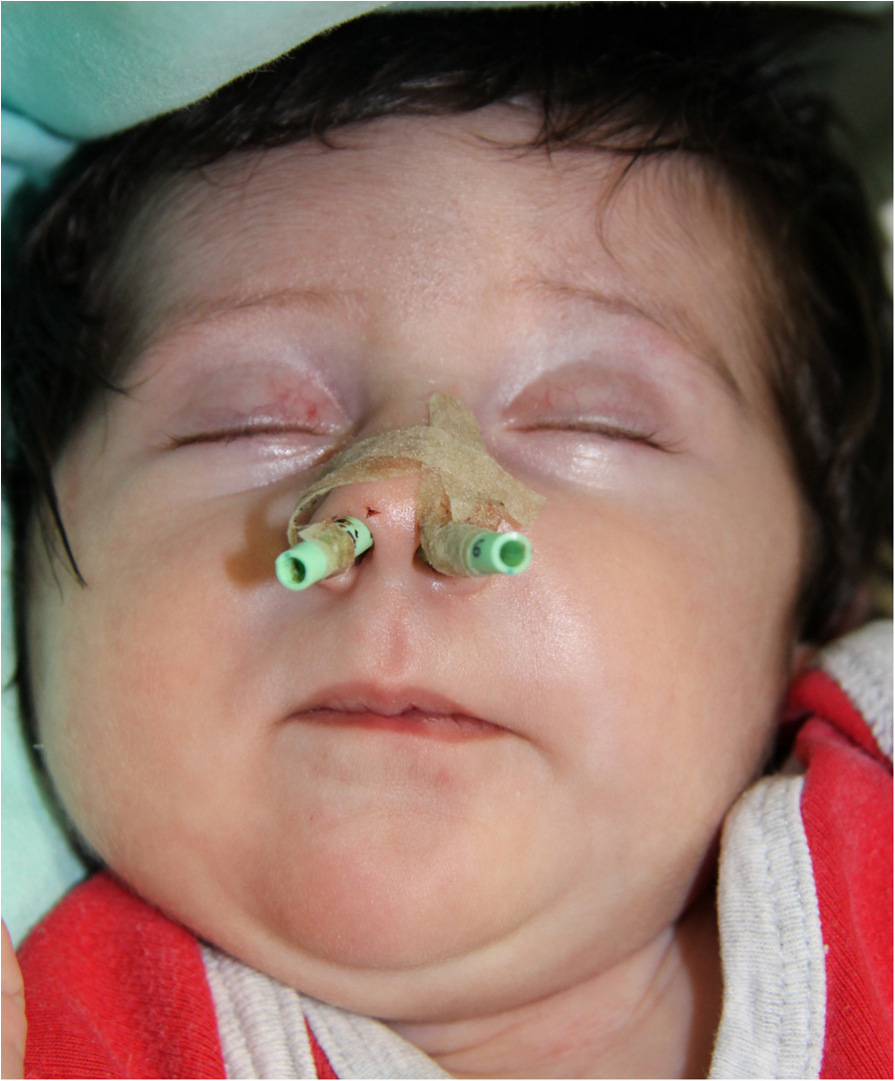
Post-operative picture showing the nasal silastic stents applied fixed to the columella.

The patient retained the orotracheal tube in the neonatal postoperative intensive care unit for 1 day and was extubated without complication. The nasal stents were removed on postoperative day 14, and nasal decongestants and saline drops were continued for another 6 days. The patient was discharged with no complication on day 16 (Figure 3).

### Case 2

A full-term female infant had Apgar scores of 5 at 1 min and 9 at 5 min after birth. Her weight was 3.520 kg, length was 53 cm, and cranial circumference was 35 cm. The infant showed tirage, cornage, episodes of desaturation to 80% with spontaneous resolution, a reduced vesicular murmur, and jugular retraction. CT showed bilateral stenosis of the pyriform aperture, 5-mm stenosis of the posterior airway lumen (normal diameter in an infant, 6–7 mm), and right deviation of the nasal septum.

The infant was maintained on decongestant drops for several days with a reasonable respiratory result, but experienced difficulties in feeding and failure to thrive. Thus, we performed endo-oral pyriform stenosis enlargement at 26 days of age. In this case, debridement of the posterior nasal fossa and turbinate was not required because the mild obstruction was mainly related to the posterior part of the turbinate and was easily displaced, achieving satisfactory airway improvement. Standard nasal conformers usually employed in cleft surgery (16 × 23 × 7 mm) were applied and maintained for 3 weeks after surgery. Nasal decongestants and saline drops were continued for 6 days, then discontinued. The patient was discharged with no complication on day 12.

### Case 3

A female infant born at 37 weeks had Apgar scores of 9 at 1 min and 10 at 5 min after birth. Her weight was 2.800 kg, length was 47 cm, and cranial circumference was 31 cm. After an initial discharge from the neonatal care unit, the infant was readmitted at 34 days of age because of multiple episodes of cyanosis, dyspnea, inspiratory stridor, and difficulties in eating. CT showed bilateral stenosis of the pyriform aperture (5,7 mm of diameter) with normal choanal morphology.

**Figure 4:**
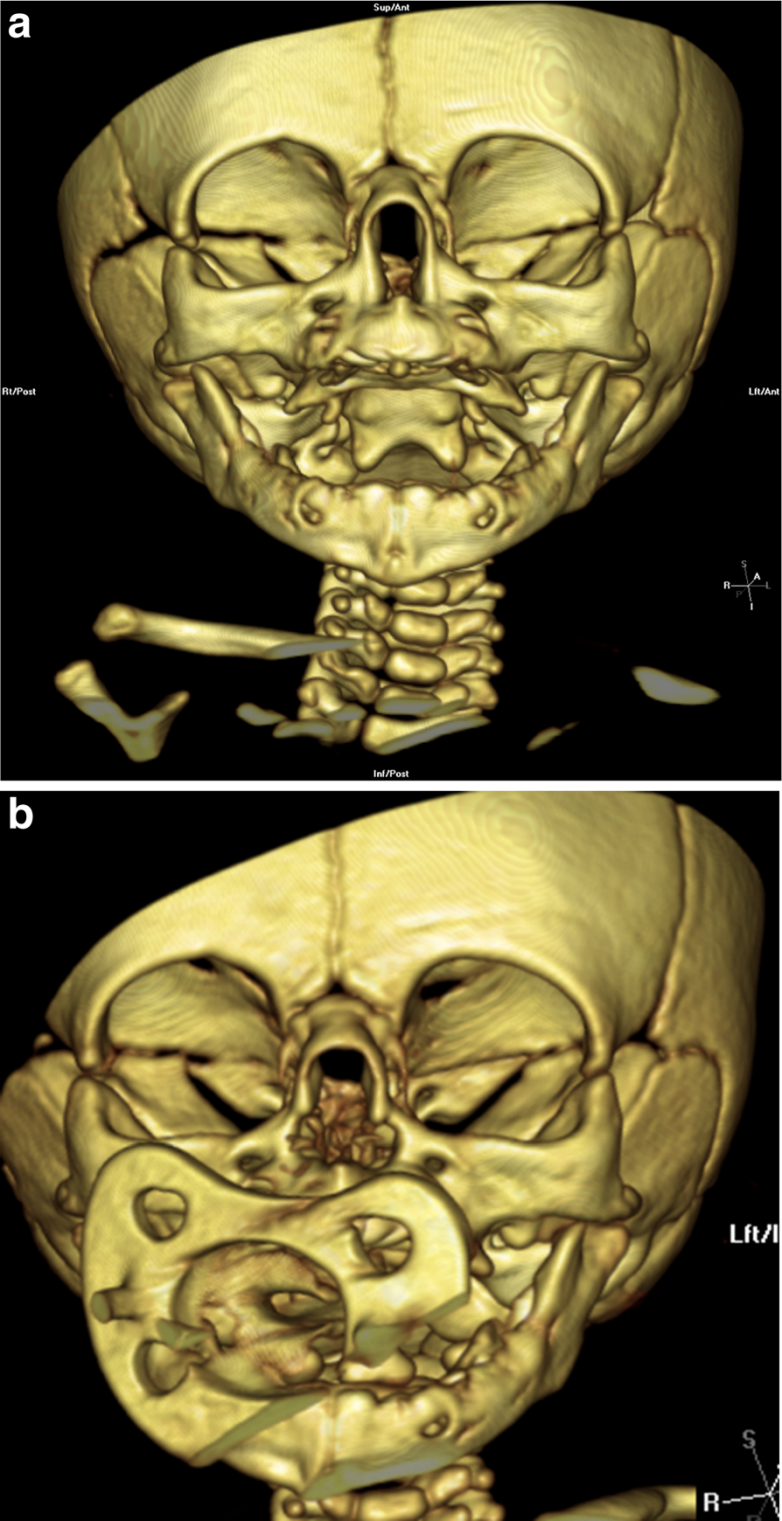
Pre-operative 3D-TC scan (a) showing the stenosis of the nasal pyriform aperture and the post-operative 3D-CT-scan (b) of the same patient showing the enlargement obtained.

**Figure 5:**
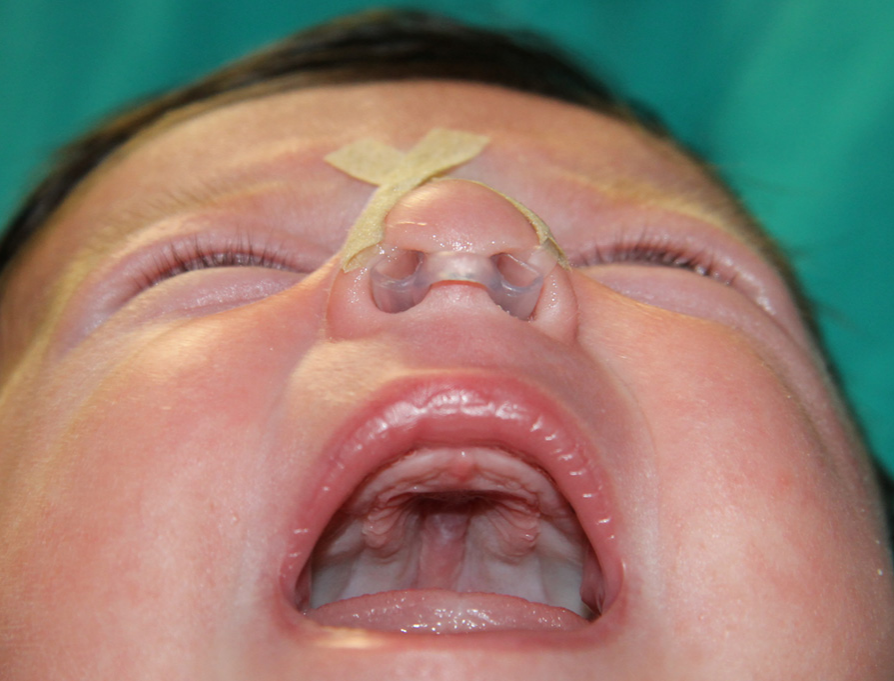
Post-operative picture showing the application of nasal conformers routinely applied in cleft patients.

Given the persistence of symptoms despite conservative treatment with local decongestants, the infant underwent surgery at 47 days of age. The pyriform opening was widened by bone drilling through an endo-oral sublabial approach, as described for cases 1 and 2 (Figure 4). Endoscopic evaluation confirmed normal anatomy of the posterior nasal fossa and, in particular, normal choanal morphology; thus, no further surgery was required. Standard nasal conformers usually employed in cleft patients (16 × 23 × 7 mm) were applied for 3 weeks to stabilize the surgical enlargement (Figure 5). The infant showed immediate postoperative improvement in respiratory function and nutrition, and was discharged with no complication on postoperative day 7.

## Results and discussion

Because neonates are obligatory nasal breathers, any condition that prevents normal nasal airflow must be diagnosed and treated correctly. CNPS is a rare clinical condition that must be considered in the differential diagnosis with other breathing difficulties of an inflammatory, traumatic, or congenital nature. The cause of this pathology is unknown, but it arises in the fourth month of fetal development because of an overgrowth of the nasal process of the maxilla and may present as an isolated condition or in association with other congenital disorders. Several studies demonstrated a relationship between CNPS and craniofacial abnormalities such as holoprosencephaly, cleft palate, and the early presence of maxillary central incisors [5,6]. This rare anatomic condition is often associated with the more common choanal atresia, which is characterized by narrowing of the posterior airways by membranous and bony tissue.

CNPS clinically presents with unspecific symptoms, such as episodes of cyanosis, inspiratory stridor, sternal retraction, thoracic asymmetry, hypoxemia, and acidosis [2,5]. These symptoms appear early in the neonatal period and can increase with exposure to upper respiratory infection. Neonatal respiratory distress is commonly related to meconium aspiration, hyaline membrane syndrome, infection, craniofacial malformation, and other congenital diseases. Thus, the exclusion of these pathologies in differential diagnosis should lead to the diagnosis of CNPS.

The diagnosis of CNPS is based on clinical evaluation, including nasal endoscopy and especially CT. The inability to pass a 5F catheter and a radiographically measured pyriform opening < 8–10 mm in a full-term infant are considered diagnostic. If holoprosencephaly is suspected by the presence of a central maxillary incisor, encephalic CT or magnetic resonance imaging should also be performed [6-8].Once the diagnosis has been confirmed, the treatment approach must take into account the severity of the clinical condition, any associated comorbidities, and the neonate’s global prognosis.

Initial management of this condition involves the establishment of a secure airway by McGovern nipple placement or endotracheal intubation, with appropriate monitoring in the intensive care unit until the exact cause and severity of nasal obstruction have been established. In cases of mild CNPS, a nonsurgical approach involving the positioning of silastic stents in the nasal cavity and the use of local decongestants is preferable [2,9]. However, the small dimensions of the nasal stents may lead to their occlusion and make daily cleaning very difficult, thus increasing the risk of obstruction and soft-tissue injury during cleaning and repositioning.

In cases of moderate or severe stenosis, the approach is surgical and involves pyriform aperture enlargement through an endo-oral sublabial approach to reshape the stenotic area with burs. This method is safe and enables good field exposure, prevents damage to the nasolabial soft tissues, and does not cause visible scarring. Morbidity is irrelevant and results are achieved immediately after surgery [10].

A transnasal approach has also been described, but in our opinion, it is not advisable in neonates because of the reduced dimensions of anatomic structures, which increase the risk of soft-tissue trauma [8].

The surgical procedure begins with bilateral exposure of the pyriform aperture to free its bony margin, leaving the mucoperiosteum intact along the nasal floor and pyriform aperture. Drilling of the nasal floor must be avoided to prevent damage to the tooth buds [2]. The aperture is considered satisfactory when it allows for the passage of a 3.5-mm endotracheal tube stent. The bony procedures should be performed anterior to the inferior turbinate to avoid nasolacrimal duct injury [2,5].

In cases of associated choanal atresia, excess membrane and bony tissue should also be removed. The use of endoscopy is currently recommended to more safely control the posterior nasal fossa and the positioning of nasal stents.

To reduce recurrence and scar-related stenosis, the use of nasal stents is recommended. The choice of device should be based on the presence of choanal atresia. In isolated CNPS, the use of short nasal conformers, such as those routinely applied in cleft patients, is preferable because of their easier management: they are more comfortable for the patient, cleaning and replacement are easier and safer, and damage related to aspiration and displacement are avoided. On the other hand, the use of longer soft silastic nasal stents (o.d. 3.96 mm, i.d. 3.0 mm) is mandatory in the presence of choanal atresia to prevent obstructive scarring in the posterior nasal area and to ensure the stability of the surgical enlargement. We typically retain the stents for about 3 weeks if choanal atresia is present and for only 6–7 days in cases of isolated CNPS [5].

## Conclusion

CNPS is a rare and potentially letal cause of airway obstruction in infants. It may occur isolated or in association with other anomalies. CNPS should be considered in the differential diagnosis whenever, in newborns, there are signs of severe nasal obstruction associated with a difficulty in passing a small catheter through the anterior nasal valve.

In cases of severe obstructions, refractory to a conservative therapy, a surgical correction of the stenosis followed by an appropriate nasal stenting is in mandatory.

The English in this document has been checked by at least two professional editors, both native speakers of English. For a certificate, please see:

http://www.textcheck.com/certificate/sTncLX.

## Consent

Written informed consent was obtained from the parents of the patient for publication of this article and accompanying images.

## Abbreviation

CNPS: Congenital nasal pyriform stenosis.

## Competing interests

We disclose any financial and personal relationship with other people or organizations that could inappropriately influence our work.

## Authors’ contributions

ES, BB, GO and AF performed surgery, ML drafted the manuscript. All authors read and approved the final manuscript.

## Role of the funding source

No Funding to disclose.
